# Study design and rationale of the ‘Balloon-Expandable Cobalt Chromium SCUBA Stent versus Self-Expandable COMPLETE-SE Nitinol Stent for the Atherosclerotic ILIAC Arterial Disease (SENS-ILIAC Trial) Trial’: study protocol for a randomized controlled trial

**DOI:** 10.1186/s13063-016-1435-9

**Published:** 2016-06-25

**Authors:** Woong Gil Choi, Seung Woon Rha, Cheol Ung Choi, Eung Ju Kim, Dong Joo Oh, Yoon Hyung Cho, Sang Ho Park, Seung Jin Lee, Ae Yong Hur, Young Guk Ko, Sang Min Park, Ki Chang Kim, Joo Han Kim, Min Woong Kim, Sang Min Kim, Jang Ho Bae, Jung Min Bong, Won Yu Kang, Jae Bin Seo, Woo Yong Jung, Jang Hyun Cho, Do Hoi Kim, Ji Hoon Ahn, Soo Hyun Kim, Ji Yong Jang, SENS-ILIAC Investigators

**Affiliations:** Department of Internal Medicine, School of Medicine, Konkuk University, Chungju, Korea; Cardiovascular Center, Korea University Guro Hospital, 80, Guro-dong, Guro-gu, Seoul, 152-703 Korea; Cardiovascular Center, Myong-Ji General Hospital, Ilsan, Korea; Department of Cardiology, Soonchunhyang University Cheonan Hospital, Cheonan, Korea; Cardiovascular Center, Kangwon University, Chuncheon, Korea; Cardiovascular Center, Severance Hospital, Seoul, Korea; Department of Cardiology, Chuncheon Sacred Heart Hospital, Chuncheon, Korea; Cardiovascular Center, Incheon Sa-Rang General Hospital, Incheon, Korea; Cardiovascular Center, Chunnam University Hospital, Kwangu, Korea; Department of Cardiology, Hanyang University Medical Center Hanmaeum Hospital, Changwon, Korea; Cardiovascular Center, Chungbuk University, Cheongju, Korea; Cardiovascular Center, Konyang University Hospital, Daejon, Korea; Cardiovascular Center, Incheon, Hanlim General Hospital, Incheon, Korea; Cardiovascular Center, Kwanju Veterans General Hospital, Kwangju, Korea; Cardiovascular Center, Seoul University Boraemea Hospital, Seoul, Korea; Department of Cardiology, St. Carollo Hospital, Suncheon, Korea; Cardiovascular Center, Soonchunhyang University Gumi Hospital, Gumi, Korea

## Abstract

**Background:**

The self-expandable COMPLETE™ stent (Medtronic) has greater elasticity, allowing it to regain its shape after the compression force reduces, and has higher trackability, thus is easier to maneuver through tortuous vessels, whereas the balloon-expandable SCUBA™ stent (Medtronic) has higher radial stiffness and can afford more accurate placement without geographic miss, which is important in aortoiliac bifurcation lesions. To date, there have been no randomized control trials comparing efficacy and safety between the self-expanding stent and balloon-expandable stent in advanced atherosclerotic iliac artery disease.

**Methods/design:**

The purpose of our study is to examine primary patency (efficacy) and incidence of stent fracture and geographic miss (safety) between two different major representative stents, the self-expanding nitinol stent (COMPLETE-SE™) and the balloon-expanding cobalt-chromium stent (SCUBA™), in stenotic or occlusive iliac arterial lesions. This trial is designed as a prospective, randomized, multicenter trial to demonstrate a noninferiority of SCUBA™ stent to COMPLETE-SE™ stent following balloon angioplasty in iliac arterial lesions, and a total of 280 patients will be enrolled. The primary end point of this study is the rate of primary patency in the treated segment at 12 months after intervention as determined by catheter angiography, computed tomography angiography, or duplex ultrasound.

**Discussion:**

The SENS-ILIAC trial will give powerful insight into whether the stent choice according to deployment mechanics would impact stent patency, geographic miss, or stent fracture in patients undergoing stent implantation in iliac artery lesions.

**Trial registration:**

National Institutes of Health Clinical Trials Registry (ClinicalTrials.gov identifier: NCT01834495), registration date: May 8, 2012

**Electronic supplementary material:**

The online version of this article (doi:10.1186/s13063-016-1435-9) contains supplementary material, which is available to authorized users.

## Background

For aortoiliac occlusive disease, iliac artery angioplasty with stenting has been a good alternative treatment modality, and endovascular revascularization can be considered without initial extensive conservative treatment [1–5]. The technical and initial clinical success of percutaneous transluminal angioplasty (PTA) of iliac artery stenosis exceeds 90 % in the literature; in addition, the technical success rate of recanalization of obstructive long lesions of iliac artery is 80–85 % with or without additional fibrinolysis [6].

As new types of stents and technical developments have been introduced, more extensive and multifocal iliac lesions have been treated with endovascular procedures [7–11]. Low morbidity and mortality as well as up to 90 % technical success rate guarantee the endovascular-first approach and the patency rates with stenting of iliac arteries compare favorably with those of surgical revascularization. In experienced centers, TransAtlantic Inter-Society Consensus (TASC) type D lesions might be also primarily treated percutaneously [12–14].

In endovascular repair for iliac lesions, unlike the infrapopliteal revascularization strategy, routine stent placement with a PTA strategy has been generally accepted [15]. It was seen in a study of 250 patients undergoing either PTA alone or PTA with provisional stenting that the provisional stenting arm was associated with more than a 2.5-fold reduction (10 vs 4 %) in immediate failures, although long-term results in the two groups were similar [16]. In a meta-analysis of iliac artery intervention studies, the risk of long-term failure was reduced by 39 % after stent placement compared with PTA and the technical success rate was higher after stent placement [7]. A study comparing angioplasty alone with angioplasty plus stenting for complex iliac artery disease showed that cumulative primary patency rates were 78 % at 1 year, with a secondary patency rate more than 80 % at 32 months, with reduction in long-term failure of 39 % when stents were used [17]. In this analysis, stenting improved the 4-year primary patency rates by approximately 10 % for both patients treated for claudication or critical ischemia, and patients treated for iliac stenosis and occlusions.

Stents for iliac artery disease are classified as self-expandable and balloon-expandable according to the mechanics of stent deployment, and as nitinol, stainless steel and others according to stent material. Currently, the choice of balloon- versus self-expandable stent is determined mainly by operator preference. Self-expanding stents are usually made of nitinol, an alloy of nickel and titanium, and stainless steel. Nitinol alloys are most commonly known for their super-elasticity and thermal shape memory [18]. Generally, self-expanding stents have greater elasticity, allowing them to regain their shape after the compression force reduces. Another advantage of self-expanding stents is their higher trackability, meaning they are easier to maneuver through tortuous vessels or pass the aortic bifurcation from the contralateral approach. Balloon-expandable stents are usually made of stainless steel. Balloon-expandable stents are characterized by much greater radial strength compared to self-expanding stents. The main advantages of balloon-expandable stents are the higher radial stiffness and more accurate placement - avoiding geographic miss, which is especially important in aortoiliac bifurcation lesions. Balloon-expandable stents generally have higher radio-opacity, which facilitates accurate placement [19]. In addition, balloon-expandable stents are generally considered more appropriate in rigid and straight lesions [20, 21].

There are some indirect comparative studies that compared balloon-expandable with self-expandable stents [22, 23], and one direct study that compared self-expandable with balloon-expandable stents in a human ex vivo [24]. The outcome of two different self-expanding stents (nitinol versus stainless steel) for the treatment of iliac artery lesions was indirectly compared in a multicenter prospective randomized trial. In the CRISP-US trial [22], the 9-month composite end-point rate was equivalent for the SMART stent versus the Wallstent (6.9 vs. 5.9 %), with low rates of restenosis (3.5 vs. 2.7 %), death (2.0 vs. 0.0 %), and revascularization (2.0 vs. 4.0 %) in both groups. Primary patency at 12 months was 94.7 and 91.1 % with the SMART stent and Wallstent, respectively. The MELODIE study demonstrated that The Express LD balloon-expandable stents were indirectly similar to the Palmatz balloon- expandable stent for primary patency (92.1 % at 6 months and 87.8 % at 2 years) [23]. Grenacher et al. reported that self-expanding stents presented considerably lower radial expansion force and lower degree of precision than balloon-expandable stents in a human cadaver bifurcation model [24]. However, there is no randomized comparative study that compares primary patency rates of the two different types of stent (self-expandable versus balloon-expandable) in patients with iliac artery disease.

Therefore, in this study, we will compare primary patency rates of self-expandable stents and balloon-expandable stents in real-world clinical practice. In addition, we would like to ascertain whether there are differences in safety, including the incidence of geographic miss rate and stent fracture rate, between both stent groups in steno-occlusive lesions, especially for aortoiliac bifurcation lesions.

## Method/design

### Study objectives, hypothesis, and design

The primary purpose of our study is to compare primary patency between self-expandable nitinol stents and balloon-expandable cobalt-chromium stents in real-world clinical practice (COMPLETE-SE™ versus SCUBA™, Medtronic, Minneapolis, MN, USA) in stenotic or occlusive iliac arterial lesions. The secondary purpose is to ascertain whether there is a difference in safety, including the incidence of geographic miss rate and stent fracture rate between both groups in patients undergoing stent implantation in iliac lesions. This is a prospective, randomized, multicenter trial to assess the efficacy and safety of the COMPLETE-SE™ versus the SCUBA™ stent by provisional stenting after balloon angioplasty for steno-occlusive iliac arterial lesions. A description of this manuscript according to the SPIRIT guidelines is presented in Additional file 1

**Fig. 1:**
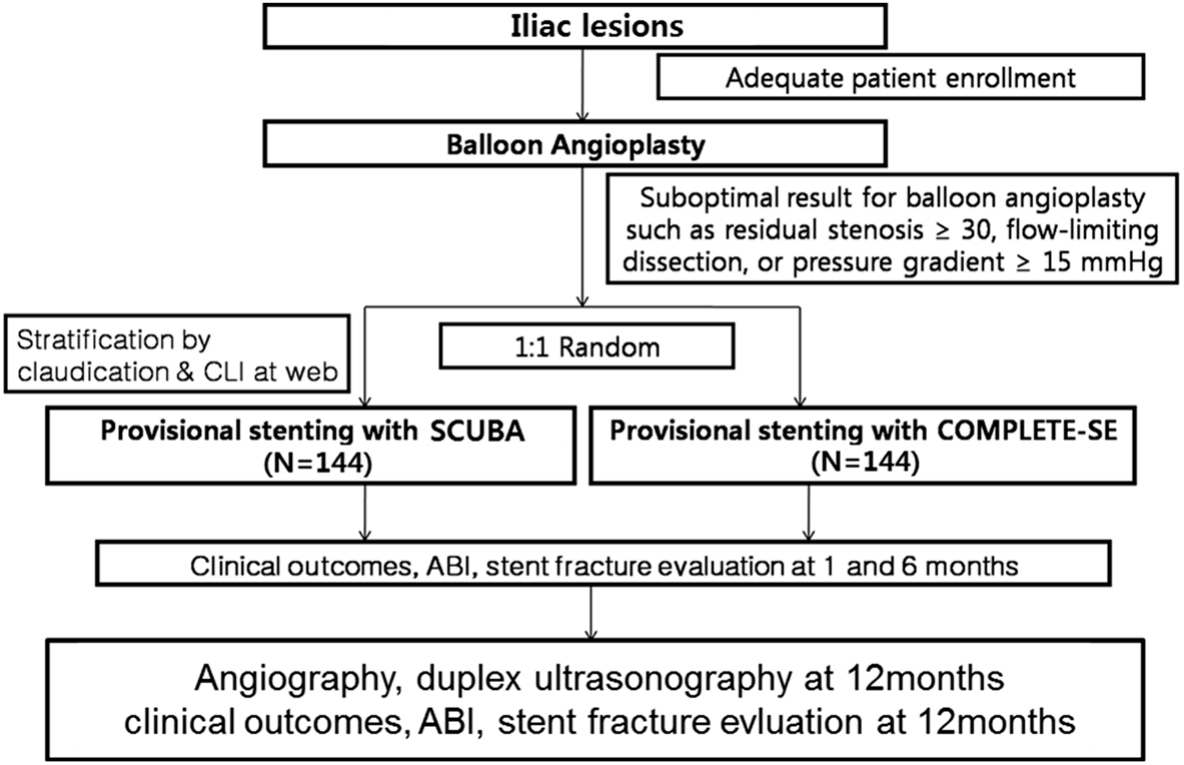
Flow chart of the enrolled patients

The protocol of the trial has been registered with the National Institutes of Health Clinical Trials Registry (registration number: NCT01834495) and a brief flow chart of the whole study is summarized in Fig. 1.

### Study population

Patients at least 20 years of age who have moderate or severe intermittent claudication or critical limb ischemia (CLI), Rutherford score of 2 to 6, except for any patient who has undergone or plans to have a major amputation) will be screened for study enrollment. Prior to inclusion, morphological examinations such as computed tomography (CT) and magnetic resonance imaging (MRI) are not necessarily needed. However, we will usually examine the CT angiography prior to the intervention. A patient will be enrolled if they meet all of the inclusion criteria and have none of the exclusion criteria. Inclusion criteria consist of clinical and anatomical criteria.

The clinical inclusion criteria are as indicated below:

1. Symptomatic peripheral artery disease with moderate to severe claudication (Rutherford score of 2 to 3)
2. Chronic CLI with resting ischemic pain (Rutherford score of 4)
3. Chronic CLI with ischemic ulcers (Rutherford score of 5 to 6)
4. Patients must provide written informed consent

The anatomical inclusion criteria are as indicated below:

1. Stenosis of more than 50 %
2. Occlusion of the ipsilateral iliac artery
3. Patent (≤50 % stenosis) ipsilateral femoropopliteal artery, or concomitantly treatable ipsilateral femoropopliteal lesions (≤30 % residual stenosis), and at least one patent (less than 50 % stenosis) tibioperoneal run-off vessel. We will not exclude patients who have undergone femoral endareterectomy. If inflow and outflow disease can be treated (except by bypass surgery), we will enroll these patients

Exclusion criteria include the following:

- Failure to provide written informed consent
- A history of major bleeding within the prior 2 months
- Known hypersensitivity or contraindication to any of the following medications: heparin, aspirin, clopidogrel, cilostazol, or contrast agent
- Acute limb ischemia
- Previous bypass surgery or stenting of the ipsilateral iliac artery
- Untreated inflow disease of the distal aorta (>50 % stenosis or occlusion)
- Patients who have undergone major amputation (amputation of above the ankle) or major amputation is planned or required
- Patients with a life expectancy of less than 1 year due to comorbidities

### Randomization and interventions

Prior to the endovascular intervention, informed consent (see Additional file 2) will be obtained from all participants by investigators. Aspirin and clopidogrel will be administered at least 12 hours before the procedure. Prior to the intervention, 70 to 100 units/kg of unfractionated heparin will be administered. Endovascular interventions will be carried out percutaneously, by placing a 6–8Fr sheath at the femoral artery via an antegrade approach or a contralateral crossover technique. In selected cases, a retrograde approach (from the distal superficial femoral artery (SFA), popliteal artery, or pedal arteries) and/or brachial approach will be allowed. Diagnostic angiography will be performed in two different views at least 30 to 45° apart to evaluate the structure of the target lesion. Femoropopliteal and tibial arteries will be visually checked for the presence of distal lesions. To document the precise location of the lesion and the site of the intervention, we will recommend the use of a ruler. In cases of total occlusion, both intraluminal and subintimal methods of recanalization will be allowed. During the procedure, the use of other special devices will be allowed; for example reentry devices OUTBACK™ catheters (Cordis Corp., Miami Lakes, FL, USA) and Offroad catheters (Boston Scientific, Natick, MA, USA), chronic total occlusion (CTO) devices Frontrunner (Cordis Corp., Miami Lakes, FL, USA) and Truepath (Boston Scientific, Natick, MA, USA), and atherectomy devices such as SilverHawk™ and TurboHawk™ (Covidien, Plymouth, MN, USA). After the successful passage of the guidewire, predilation of the target lesion with an optimally sized balloon will be performed prior to stent implantation. Recommended minimal balloon dilation time is 120 seconds. Then, if there is a residual pressure gradient of ≥10 mmHg, residual stenosis of ≥30 %, or flow-limiting dissection, the balloon angioplasty results will be considered as suboptimal results. Subsequently, web-based randomization for stent selection will be performed. Patients will be randomly allocated to one of two groups, the balloon-expandable or self-expandable stent group, according to the stent deployment method. Random allocation of the patients will be performed via a web-based computerized program separately managed at the Cardiovascular Intervention Research Institute (CIRI), Korea University Guro Hospital, Seoul, Republic of Korea. Patients will be randomized in a 1:1 manner according to two different stents (SCUBA™ versus COMPLETE-SE™). The stents will be implanted to extend 10 mm proximally and distally from the margins of the target lesion with luminal narrowing of ≥50 %. A proper stent size will be selected after review of the baseline angiography results. If the stent is randomized to the COMPLETE-SE™, a stent with a diameter 1 to 2 mm larger than that of the true vessel lumen will be selected. However, if the stent is randomized to the SCUBA™ stent, a stent of equal size to the reference vessel diameter will be selected to prevent iliac artery injury. Spot stenting or full lesion coverage will be decided at the physician’s discretion. When multiple stents are required, the margins of the stents should be overlapped by at least 10 mm. Adjuvant postdilation after the stenting will be performed strictly within the stented segment, with up to 10 % oversizing of the postdilation balloon.

The self-expandable COMPLETE-SE™ stent has three distinct crown configurations, and an alternating connection pattern minimizes crown-to-crown interaction and provides optimal flexibility, foreshortening, and lesion coverage for each stent size. The triaxial deployment design includes an inner shaft, a retractable sheath, and a stabilizing sheath, which reduces friction and allows the retractable sheath to move back freely. This decreases the amount of force required to deploy the stent, making deployment easy and accurate. Diameter and length of self-expandable COMPLETE-SE™ stents are 5–10 mm and 20, 40, 60, 80, 100, 120, and 150 mm, respectively. Balloon-expandable SCUBA stents are composed of thin cobalt-chromium struts, unlike other stainless steel stents, and have a closed-cell design for tight scaffolding and appropriate radial strength in iliac lesions. Diameter and length of balloon-expandable SCUBA™ stents are 6–10 mm and 20, 30, 40, and 60 mm, respectively. The entire stent size (the COMPLETE-SE™ and SCUBA™ stent) is now 6-Fr sheath compatible. We will use either a 0.035” or 0.018” wire system and implant either a SCUBA™ or COMPLETE-SE™ stent. All ipsilateral femoropopliteal arterial lesions should be treated with angioplasty and/or stenting concomitantly and residual stenosis should be less than 50 %. Treatment of tibioperoneal lesions is recommended only in cases of CLI. There should exist at least one patent (<50 % stenosis) tibioperoneal run-off vessel with good antegrade flow. A final angiography will be performed after the intervention in both groups, with the use of the same angles and magnifications used at the baseline angiograms. Simultaneously, reference vessel diameter (RVD), minimal luminal diameter (MLD), percent diameter stenosis, and acute gain should be measured. The RVD will be obtained from averaging 5-mm segments proximal and distal to the lesion. Technical success will be defined as successful access, deployment of the stent, and less than 30 % diameter residual stenosis after the revascularization. For postprocedural medication, aspirin 100 mg and clopidogrel 75 mg will be administered once daily for at least 12 months. Cilostazol would be permitted as the third antiplatelet at the physician’s discretion. After enrollment and index procedure, clinical follow-up will be planned at 1, 6, and 12 months to evaluate clinical outcomes and ankle-brachial index (ABI) score. In addition, a follow-up catheter angiography, CT angiography, or duplex ultrasound will be recommended to all patients at 12 months, according to local clinical practice. The investigators will be urged to follow up on patients, either by office visits or by telephone contacts, as necessary. Patient adherence to the study drug and side effects will be checked and monitored at every outpatient visit. The decision of drug discontinuation will be discussed and checked at the physician’s recommendation.

Participants have the right to withdraw from the study at any time for any reason. The principal investigator also has the right to withdraw patients from the study in the event of protocol violations, administrative reasons, or other reasons, e.g., the participant is no longer being treated at a hospital included in the study. It is understood by all concerned that an excessive rate of withdrawals can render the study uninterpretable; therefore, unnecessary withdrawal of patients should be avoided.

### Outcome measures

The primary end point of this study is the patency rate (stenosis of at least 50 % of the luminal diameter) on catheter or CT angiography or <2.5 of peak systolic velocity ratio (PSVR; peak systolic velocity within the area of stenosis divided by peak systolic velocity in a normal adjacent proximal artery segment) in the treated segment at 12 months after the intervention.

The secondary end points are as follows: (1) geographic miss rate (mild, moderate, severe); geographic miss refers to stent deployment to any area of the vessel that was intended to be treated with the stent but was not covered, or protrusion due to stent jumping, and/or elongation. Geographic miss rate is classified as 5–15 mm (mild), 15–25 mm (moderate), >25 mm (severe), or additional stent deployment over the stent deployment area that was not covered, or protrusion from the intended stent deployment area; (2) incidence of stent fracture; (3) limb salvage rate free of above-the-ankle amputation; (4) sustained clinical improvement rate at 12-month follow-up; (5) repeated target lesion revascularization (TLR) rate; (6) repeated target extremity revascularization (TER) rate; (7) total reocclusion rate; (8) comparison of angiographic variables including late loss and binary restenosis rate; (9) ABI at 12 months; (10) the rate of major adverse cardiovascular event (MACE), defined as composite of all-cause death, myocardial infarction, repeat percutaneous coronary intervention (PCI), and stroke at 12 months.

The detailed definitions are summarized in the Appendix.

#### Sample size calculation

Although patency definitions varied across trials, 1-year patency rates in aortoiliac lesions were approximately 90 % on average from previous studies including CRISP-US and MELODIE [22, 23]. In the case of the SCUBA™ stent, the 9-month patency rate for the SCUBA™ stent was 99.2 % in the ACTIVE study - a prospective, multicenter, single-arm study that defines the midterm efficacy of a next-generation cobalt-chromium iliac stent for the treatment of de novo and restenotic lesions in iliac arteries [25]. However, a different definition - a ratio of <2.4 between the duplex peak systolic velocity in the treated vessel segment and the reference systolic velocity - was used, unlike the more common definition. There are limited data on the 1-year primary patency rate of the SCUBA™ stent. Based on the MELODIE trial [23], and the European multicenter iliac stent trial [26], the 1-year primary patency of the SCUBA™ stent was estimated to be 88 %, and the COMPLETE-SE™ stent was estimated to be 94.7 % in the CRISP-US trial. Therefore, the self-expandable COMPLETE-SE™ stent group will be the control group, and the balloon-expandable SCUBA™ stent group will be the experimental group.

Patency rate will be defined as absence of occlusion and absence of >50 % stenosis in the treated segment by quantitative coronary angiography (QCA) or if invasive angiography is not performed, PSVR ≤2.5 when evaluated with duplex ultrasound. On the basis of previous studies [22, 23], we hypothesize that the expected patency rate after stent deployment at 1 year would be 90 % for the self-expandable stent group, and 88 % for the balloon-expandable stent group, respectively. The predetermined noninferiority margin δ is a 10 % difference between treatment groups. There was clinical consensus that this noninferiority margin would be acceptable if safety is maintained and patients are treated more easily, regardless of stent classification. The power analysis is based on a noninferiority principle. The statistical significance level is 2.5 % at one side, the power of the test is set as 80 %, and the randomization ratio is set as 1:1. The test for proportion and the chi-square method will be used. Using standard sample size formula, it was calculated that 120 patients per group are needed for a total of 288 patients, after accounting for a 20 % dropout rate.

### Statistical analysis

The IBM SPSS 20.0 (IBM Corp., Armonk, NY, USA) statistics program will be used for all analysis. Categorical variables will be expressed as delivery rate when comparing baseline features between the SCUBA™ and COMPLETE-SE™ stent groups and for categorical variables, comparisons between groups will use the chi-square or Fisher’s exact test. Continuous variables will be expressed as mean ± standard deviation, and for continuous variables, comparisons between groups will use the Student’s *t* test. The primary and secondary end points will be analyzed using both intention-to-treat analysis (all subjects assigned to a treatment group) and per protocol analysis (only subjects who completed the treatment protocol). Noninferiority will be considered proven if conclusions drawn from the intention-to-treat and per protocol analyses are consistent. Balloon-expandable stent patency will be judged as noninferior to that of the self-expandable stent when the lower limit of the 95 % CI of the difference between the proportions of patent patients at 1 year in both groups is higher than -δ = −10 % At the end of the 12 months, cumulative restenosis rates will be calculated. For a secondary end point, we will summarize with descriptive statistics and compare between treatment groups using the Student’s *t* test, chi-square test, or Fisher’s exact test. Also, clinical outcomes such as revascularization of the target vessel, death, major cardiovascular events including myocardial infarction and cerebral infarction, and limb salvage rates will be analyzed using Kaplan-Meier survival estimates and a comparison of the groups will be performed using the log-rank test. A *P* value <0.05 will be considered to be statistically significant. In order to evaluate the risk factors for recurrence, defined as restenosis or total occlusion, univariate and multivariate logistic regression analysis will be performed. The present research has concluded that there will be a very low chance of missing data to cause bias, and therefore, missing data for the major end points will be excluded from the analysis data.

### Trial organization

#### Executive committee

The Executive Committee will be composed of the study chairperson and selected members among the investigators. This committee is responsible for overseeing the administrative progress of the study and will approve the final trial design and protocol issued to the Data and Safety Monitoring Board (DSMB) and the clinical sites. This committee will also be responsible for reviewing the final results, determining the methods of presentation and publication, and selection of secondary projects and publications by members of the Steering Committee. The Executive Committee also holds the right to modify or stop the study prematurely based on recommendations from the DSMB.

#### Data Safety Monitoring Board (DSMB)

The frequency of the DSMB meetings will be determined prior to study commencement. Additionally, the DSMB may call a meeting at any time if there is reason to suspect that safety is an issue. The DSMB is responsible for making recommendations regarding any safety or compliance issues throughout the course of the study and may recommend to the Executive Committee to modify or stop the study. However, all final decisions regarding study modifications rest with the Executive Committee. All cumulative safety data will be reported to the DSMB and reviewed on an ongoing basis throughout enrollment and follow-up periods to ensure patient safety. Every effort will be made to allow the DSMB to conduct an unbiased review of patient safety information. All DSMB reports will be made available to the appropriate agencies upon request, but will otherwise remain strictly confidential. Prior to the DSMB’s first review of the data, the DSMB charter will be drafted. The DSMB will develop a consensus understanding of all trial end points and definitions used in the event of an adjudication process. All DSMB reports will remain strictly confidential, but will be made available to the regulatory body upon request.

#### Clinical Event Adjudication Committee (CEAC)

The Clinical Event Adjudication Committee (CEAC) is comprised of interventional and noninterventional cardiologists who are not involved in the study. The CEAC is charged with the development of specific criteria used for the categorization of clinical events and clinical end points in the study, which are based on protocol. At the onset of the trial, the CEAC will establish explicit rules outlining the minimum amount of data required and the algorithm to be followed in order to classify a clinical event. All members of the CEAC will be blinded to the primary results of the trial. The CEAC will meet regularly to review and adjudicate all clinical events. The CEAC will also review and rule on all deaths that occur throughout the trial.

### Data coordination and site management

Data coordination and site management services will be performed at the Cardiovascular Center of the Korea University Guro Hospital.

### Timeline

A SENS-ILIAC trial timeline description is presented in Additional file 3.

## Discussion

A primary endovascular approach may be attempted in all patients with peripheral artery obstructive disease including iliac lesions. In performing endovascular repair for iliac artery lesion, different strategies and types of stents are needed, as described above. There is no general consensus on which stents, balloon-expandable or self-expandable stents are the most appropriate for specific lesion of the iliac artery.

The factors associated with patency after stent implantation in iliac lesion have been known to be various: patient characteristics, lesion characteristics, and so on. Recently, various self-expandable nitinol stents have been manufactured with improved radial force, reduced stent deformity or fracture, and convenient deployment systems. Currently, self-expandable nitinol stents have been the most widely used.

The next-generation balloon-expandable stent has been developed. The first Assurant® balloon-expandable stent system (Medtronic, Inc., Santa Rosa, CA, USA) made from a cobalt-chromium alloy is to be approved by the Food and Drug Administration (FDA) for the treatment of narrowed iliac arteries. The mechanical properties of the cobalt-chromium alloy allow the device to have thinner struts and a low profile, which provide increased flexibility while maintaining radial strength and fluoroscopic visibility [27]. In the ACTIVE study [25], the balloon-expandable Assurant® cobalt-chromium iliac stent demonstrated primary patency at 9 months of 99.2 %, device and lesion success were each 97.5 % (155/159), and the procedural success was 96.7 %.

In real-world clinical practice, physicians tend to deploy self-expanding stents in the mid-iliac area due to severe angulation and tortuosity for fear of iliac artery injury including extravasation, dissection, or rupture following high-pressure balloon inflation with longer balloon-expandable stents. It will be of interest to see the safety rate of balloon-expandable stents in the tortuous mid-iliac artery.

However, because of the possibility of ‘geographic miss’- by stent elongation or jumping during stent deployment - which may cause stent fracture, in-stent restenosis, and contralateral artery flow limitation, the balloon-expandable stent has been recommended for accurate stent deployment, especially in bifurcation lesion, including common iliac artery ostium. It will also be important to compare the geographic miss rate between the balloon-expandable SCUBA stents and self-expandable COMPLETE-SE™ stents in iliac artery lesion, especially aortoiliac bifurcation lesion.

One of the significant limitations to previous studies is that they were nonrandomized studies of relatively small sample size, indirect comparisons, or expert opinions. To date, a multicenter, randomized controlled trial for direct comparison of efficacy and safety between self-expandable stents and balloon-expandable stents has not been done.

This trial was designed to evaluate the efficacy and safety between self-expandable COMPLETE-SE™ versus balloon-expandable SCUBA™ stents in patients undergoing stent implantation in iliac artery lesions and will provide a rationale for which choice of stent will be optimal in iliac steno-occlusive disease, particularly with regard to accurate lesion location.

In conclusion, we still do not know whether there is a difference in primary patency, incidence of geographic miss, and stent fracture between self-expandable nitinol stents and balloon-expandable cobalt-chromium stents (COMPLETE-SE™ versus SCUBA™) in stenotic or occlusive iliac arterial lesion. We hope to address these issues in the SENS-ILIAC trial where we have enrolled a large unselected population of patients treated with stent implantation for significant iliac arterial disease.

## Trial status

This study is ongoing and is recruiting.

## Abbreviations

ABI, ankle-brachial index; CEAC, Clinical Event Adjudication Committee; CIRI, Cardiovascular Intervention Research Institute; CLI, critical limb ischemia; CT, computed tomography; CTO, chronic total occlusion; DSMB, Data and Safety Monitoring Board; FDA, Food and Drug Administration; Hct, hematocrit; Hgb, hemoglobin; MACE, major adverse cardiovascular event; MLD, minimal luminal diameter; PCI, percutaneous coronary intervention; PSVR, peak systolic velocity ratio; PTA, percutaneous transluminal angioplasty; QCA, quantitative coronary angiography; RVD, reference vessel diameter; SFA, superficial femoral artery; TASC, TransAtlantic Inter-Society Consensus; TER, target extremity revascularization; TLR, target lesion revascularization

## Appendix

### 1. End points definitions

- Primary patency: defined as stenosis of at least 50 % of the luminal diameter according to the worst angiographic view at the narrowest site within the treated segment plus the 10-mm segments proximal and distal to the treated segment by catheter angiography or CT angiography; PSVR ≥2.5 (PSVR = peak systolic velocity within the area of stenosis divided by peak systolic velocity in a normal adjacent proximal artery segment) or total occlusion by duplex ultrasound
- Geographic miss according miss grade (mild, moderate, severe): geographic miss is classified as 5–15 mm (mild), 15–25 mm (moderate), >25 mm (severe), or additional stent deployment over the stent deployment area that was not covered, or protrusion from the intended stent deployment area
- Stent fracture rate according fracture grade (minor, moderate, severe), [28] plain X-ray examinations for the stent fracture are used or performed using at least two different angulations (30 to 45° difference) and the highest available magnification. Stent fracture is classified as minor (single strut fracture), moderate (fracture of more than two struts), or severe (complete separation of stent segments)
- Target lesion revascularization (TLR): defined as any repeat percutaneous intervention of the target lesion or bypass surgery of the target vessel performed for restenosis because of a return of ischemic symptoms, decrease of at least one Rutherford category, decrease in the ankle brachial index of >0.15, or restenosis of >50 % as measured by catheter angiography. The target lesion is defined as the treated segment from 10 mm proximal to the stent and to 10 mm distal to the stent
- Target extremity revascularization (TER): defined as any repeat percutaneous intervention of the target extremity or bypass surgery of the target extremity performed for restenosis because of a return of ischemic symptoms, decrease of at least one Rutherford category, decrease in the ankle brachial index of >0.15, or restenosis of >50 % as measured by catheter angiography
- Limb salvage: defined as survival free of amputation. Amputation is defined as above-ankle amputation of the index limb
- Sustained clinical improvement rate [29, 30] defined as persistent ABI value ≥0.15 and persistent improvement of ≥1 class according to Rutherford throughout follow-up when compared with baseline without the need for repeat TLR in surviving patients. Late loss: defined as a change in MLD from final angiogram to follow-up
- Major adverse cardiac events (MACE): defined as composite of all-cause death, myocardial infarction, cerebrovascular stroke, and target lesion revascularization

### 2. Immediate outcomes

- Acute lesion success: defined as a residual stenosis of ≤30 % without flow-limiting dissection in the worst angiographic view
- Definition of suboptimal result: residual pressure gradient of >15 mmHg, residual stenosis of >30 %, or flow-limiting dissection
- Acute hemodynamic success: defined as a ≥0.15 improvement in the ankle-brachial index from preprocedure to immediately postprocedure (discharge)
- Clinical success: defined as an improvement of baseline symptoms by at least one Rutherford category that was sustained throughout follow-up with no additional intervention

### 3. QCA data definition and variables

- Reference vessel diameter (obtained from averaging 5-mm segments proximal and distal to the lesion)
- Minimal luminal diameter (MLD)
- Acute gain (change in MLD from baseline to postintervention)
- Late loss (change in MLD from the final angiogram to follow-up)
- Percent diameter stenosis
- Total reocclusion rate
- Patency (<50 %) rate

### 4. Ankle-brachial index (ABI) is calculated as following:

- Measurement of highest systolic pressure in both arms
- Measurement of systolic pressure in both legs
- Use highest ankle pressure (dorsalis pedis or posterior tibialis) for each leg
- Calculate ratio of each ankle to brachial pressure by dividing each ankle by highest brachial pressure.

Bleeding/hemorrhagic complications

### 5. An episode of bleeding is defined by the Thrombolysis in Myocardial Infarction (TIMI) criteria as:

- Major: overt clinical bleeding (or documented intracranial or retroperitoneal hemorrhage) associated with a drop in hemoglobin (Hgb) of greater than 5 g/dl (0.5 g/l) or in hematocrit (Hct) of greater than 15 % (absolute) ^*^Note: a patient who experiences an intracranial hemorrhage should be considered to have a major hemorrhage
- Minor: overt clinical bleeding associated with a fall in hemoglobin of 3 to less than or equal to 5 g/dl (0.5 g/l) or in hematocrit of 9 % to less than or equal to 15 % (absolute)
- None: no bleeding event that meets the major or minor definition

^*^Note: in calculating the fall in hemoglobin or hematocrit, a transfusion of whole blood or packed red blood cells is counted as 1 g/dl (0.1 g/l) hemoglobin or 3 % absolute in hematocrit. This would be in addition to the actual fall in hemoglobin or hematocrit.

^*^To account for transfusion, hemoglobin and hematocrit measurements will be adjusted for any packed red blood cells or whole blood given between baseline and posttransfusion measurements. A transfusion of one unit of blood will be assumed to result in an increase of 1 g/dl in Hgb or of 3 % in Hct. Thus, to calculate the true change in Hgb or Hct if there has been an intervening transfusion between two blood measurements, the following calculations should be performed: Hgb =[baseline Hgb-posttransfusion Hgb] + [number of transfused units] Hct = [baseline Hct-posttransfusion Hct] + [number of transfused units × 3].

The following will be classified as “instrumented” major bleeding that is considered to be associated with the catheterization laboratory visit:

- Major percutaneous entry site: bleeding occurred at the percutaneous entry site during or after the catheterization laboratory visit until discharge. The bleeding should require a transfusion and/or prolong the health care facility stay, and/or cause a drop in Hgb >5 g/dl
- Bleeding at the percutaneous entry site can be external, or a hematoma >10 cm for femoral access or >2 cm for radial access, or >5 cm for brachial access
- Major retroperitoneal, gastrointestinal, and genital/urinary: bleeding occurred during or after the catheterization laboratory visit until discharge. The bleeding either requires surgical intervention (e.g., to relieve nerve compression), and/or requires a transfusion and/or prolongs the health care facility stay, and/or causes a drop in hemoglobin >5.0 g/dl
- Major other/unknown: bleeding occurred at other or unknown locations during or after the catheterization laboratory visit until discharge. The bleeding should require a transfusion and/or prolong the health care facility stay, and/or cause a drop in Hgb > 5 g/dl

Stent size and compatibility: compatible wire system: 0.035” or 0.018” wire system. Diameter and length of self-expandable COMPLETE-SE™ stent are 5–10 mm and 20, 40, 60, 80, 100, 120, and 150 mm, respectively. Diameter and length of balloon-expandable SCUBA™ stent are 6–10 mm and 20, 30, 40, and 60 mm, respectively.

## Additional files

Additional file 1: SPIRIT checklist. (DOCX 60 kb) Additional file 2: Consent form. (DOCX 28 kb) Additional file 3: The schedule of enrollment, interventions, and assessments. (DOCX 22 kb)
