# Supplementary material for: Study design and rationale of the ‘Balloon-Expandable Cobalt Chromium SCUBA Stent versus Self-Expandable COMPLETE-SE Nitinol Stent for the Atherosclerotic ILIAC Arterial Disease (SENS-ILIAC Trial) Trial’: study protocol for a randomized controlled trial
Source: Trials. 2016 Jun 25;17:302. doi: 10.1186/s13063-016-1435-9 (PMC4920989; doi:10.1186/s13063-016-1435-9)
Supplement: Additional file 2: — Consent form. (DOCX 28 kb) [file 13063_2016_1435_MOESM2_ESM.docx]

**피험자 설명문**

Consent Form of SENS-ILIAC study

**연구 과제명**

**Study title**

동맥경화성 장골 동맥 질환에서 풍선 확장 **Cobalt Chromium** 스텐트인 SCUBA™ 스텐트와 자가 팽창 nitinol 스텐트 COMPLETE-SE™ 스텐트의 효과를 비교하기 위한 전향적, 다기관 공동, 무작위 배정, 비교 연구

**연구 배경 및 목적**

**What is the purpose of this study?**

우리나라에서는 고령 인구의 증가, 높은 흡연율, 고혈압, 당뇨, 동맥경화증 등 성인병이 증가하고 있습니다. 이러한 이유로 연관되어 뇌졸증, 협심증, 심근경색 등 혈관들이 좁아지거나 막히는 질환 빈도 역시 높아지고 있습니다. 최근에는 특히 흡연, 당뇨와 연관되어 발생하는 다리 통증이나 다리 궤양 및 궤사 환자 문제가 사회적으로, 의학적으로 대두되어 이전보다 보다 적극적으로 환자에 대한 교육 및 치료가 발전하고 있는 상황입니다.

다리 혈관 질환에 대한 치료로는 전통적으로 약물 치료 및 외과적인 수술 치료 방법이 사용되어 왔으나, 기술 및 기구가 발전함에 따라 최근 10년 사이에 전신 마취나 절개 없이 가는 관을 이용한 풍선혈관 확장술 및 스텐트 삽입술 등 중재 시술이 활발이 이루어 지고 있습니다. 이에 따라 다리 동맥인 장골 및 대퇴 슬와 동맥에 대한 중재시술 치료는 많이 보편화되었습니다.

장골 동맥 질환에서 스텐트 삽입치료는 고식적인 풍선 혈관 성형술에 비해 우월한 결과를 나타내어 보편적으로 사용되고 있습니다. 하지만 장골 동맥입구 병변, 복잡한 병변의 경우 아직까지 재발율이 높고, 각기 다른 방식의 스텐트 삽입에 따른 전향적 임상 연구는 부족한 실정입니다. 동시에 다른 종류의 스텐트에서 재발율에 대한 차이를 알아보기 위한 전향적 임상 연구는 거의 없습니다. 따라서 본 연구는 장골 동맥 질환에서 현재 임상에서 널리 사용되고 있는 자가 팽창형 스텐트인 COMPLETE-SE^TM^ 스텐트와 풍선 확장형 스텐트인SCUBA^TM^ 스텐트에서 재발율의 차이가 있는지를 알아보고자 계획되었습니다.

**검증되지 않은 연구의 실험적인 측면 설명 및 확인에 대한 내용**

**What are the risks?**

장골 동맥 질환에서 풍선 혈관 성형술과 스텐트 삽입술은 보험 적용을 받고 있으며 현재 임상에서 일반적으로 사용되는 표준 치료 방법입니다. 현재까지 치료 가이드라인에 따르면 스텐트 삽입술은 풍선 혈관 성형술의 결과가 만족할 만한 수준이 되지 못할 때 사용됩니다. 장골동맥 병변에서 니티놀 스텐트 삽입후 1년 재발율은 병변의 특성이 길고 복잡할수록 재발율이 높고, 이와 관련된 임상적, 해부학적 요인 또한 다양합니다. 현재까지 연구에 따르면 니티놀 금속 스텐트의 경우 경우 1년 개존률이 90% 정도로 보고되고 있고 또한 몇몇 연구에 따르면 스텐트의 종류에 따라 다를 수 있다고 보고 되고 있습니다. 국내에 시판되어 사용되고 있는 자가 팽창형 니티놀 금속 스텐트는 여러 종류가 있는데, 그 중 COMPLETE-SE^TM^ 스텐트와 풍선 확장형 cobalt chromium SCUBA^TM^ 스텐트는 보편적으로 사용되고 있고 현재까지 후향적 및 등록 관찰 연구에서 재발율이 적은 스텐트로 알려져 있습니다. 이 두가지 스텐트는 금속 스텐트라는 점에서는 동일하지만 스텐트 삽입 방법에 있어 차이를 보이고 있습니다. 아직까지 자가 팽창형 스텐트와 풍선 확장형 스텐트 종류의 니티놀 금속 스텐트에서 개존률에 대한 차이가 있는지에 대한 직접적인 비교 연구가 없는 실정입니다.

**실시기관, 시험책임자, 참여하는 대상 환자 수 및 예상되는 연구 기간**

**Study sites, Principle investigators, Participated patients, Study duration**

본 연구는 국내 종합병원, 대학병원에서 공동으로 실시하며 연구의 총책임자는 고려대학교 구로병원 순환기내과 나승운 교수님이며, 시험 공동 책임자는 건국의대 충주병원 심장내과 최웅길 교수님입니다. 공동연구기관으로 고려의대 구로병원, 순천향의대 천안병원, 관동의대 명지병원등이 참여하고 있습니다. 본 연구의 연구기간은 2012년 9월부터 2015년 8월까지 36개월 정도이며 대상환자수는 280명입니다.

**연구 방법**

**Study Methods**

치명적 하지 허혈 환자 또는 파행증 환자중 장골 동맥 질환으로 혈관 중재 시술을 시행 받은 환자 중 선정기준과 제외기준을 검토하여 대상을 정하고 연구의 목적과 방법 및 잠재적 부작용과 이익에 대해 환자에게 충분한 설명을 하고 이에 동의할 경우 본 연구에 등록됩니다. 선정 기준은 20세 이상, 중등도 내지 중증 파행증 또는 만성 하지 허혈에 의한 안정시 하지 통증, 궤양, 괴사의 증상이 있는 장골 동맥 질환 환자 중 연구에 동의한 경우입니다. 제외 기준은 급성 하지 허혈 환자이거나 2개월 내 다량 출혈이 있었던 경우, 심각한 다른 동반 질환이 있거나 잔여 기대 생존기간이 1년 미만인 경우, 병변이 있는 동측 장골 동맥에 스텐트를 삽입했거나 수술한 기왕력이 있는 경우, 발목위 하지 절단술을 시행받은 경우, 20세 미만 또는 85세 이상인 경우, 헤파린, 아스피린, 클로피도그렐 및 조영제 등에 과민 반응이 있거나 금기증이 있는 경우, 임산부 또는 임신 가능성이 있는 경우와 혈관 조영 결과 본 연구에 적합하지 않는 소견이 발견된 경우입니다. 연구에 참여를 동의하신 환자는 등록 후 컴퓨터를 통한 무작위 추출방식으로 COMPLET-SE^TM^ 스텐트와 SCUBA^TM^ 스텐트 삽입군 두 군으로 나누어져 스텐트 삽입술을 시행하게 됩니다. 이후 1년 동안 1개월, 6개월 병원에 내원하여 발목 상완 지수와 스텐트 골절에 대한 평가 및 임상적인 추적 관찰을 시행하며 12개월 째에 혈관 개통 유지를 확인하게 위해 혈관 조영술을 시행받게 됩니다. 총 관찰기간은 등록 후 12개월이며 이후에도 추가적인 관찰이 진행될 수 있습니다.

**치료(약물 또는 의료기기)의 예견되는 효과**

**What benefit will I receive?**

본 연구에서 환자는 어떤 군에 속하게 되더라도 표준적인 혈관 중재 시술 치료를 받게 됩니다. COMPLETE-SE^TM^ 스텐트와 SCUBA^TM^ 스텐트는 현재 국내에서 일반적으로 사용하고 있는 스텐트로 보험 적용을 받고 있습니다. 또한 두 스텐트 모두 후향적 및 등록 관찰 연구에서 효과가 증명된 스텐트입니다. 하지만 이 두 스텐트에서 어느 것이 더욱 효과적인지에 관해 직접 비교한 연구는 없습니다. 따라서 연구에서 보고자 하는 것은 두가지 다른 종류의 풍선 확장형 스텐트와 자가 팽창형 스텐트를 직접 비교하여 환자에게 어느 것이 이익이 될지에 관한 것입니다.

**피험자 준수사항**

**What would I have to do?**

본연구에 참여하시는 환자분이 준수해야 하는 사항은 임상 연구에 참여하지 않는 경피적 관상 동맥 중재술을 시행받는 환자와 같습니다

**약물 또는 의료기기의 위험성, 가능한 약물 또는 의료기기의 이상반응에 대한 설명**

**Devices and drugs induced adverse events**

본 연구에서 사용하는 모든 치료 방법은 모두 식약청 승인 후 임상에서 널리 사용되고 있으므로 본 연구로 인하여 추가적으로 발생되는 이상반응은 없을 것입니다. 중재 시술 치료 시 환자분의 불편 발생이 최소화 할 수 있도록 의료진은 최선을 다할 것입니다. 각 약제에 과민반응이 있거나 시술 위험성이 높은 환자는 연구에서 배제 되도록 하고 있습니다.

**대체 치료법(해당 질환에 대한 타 치료 방법의 유무 및 내용)**

**Alternative treatment**

기본적으로 장골 동맥 질환에 대한 일반적인 약물 치료는 두 군 모두에서 동일하게 시행될 것입니다. 약물 치료만 필요한 경우에는 연구에 포함되지 않을 것입니다. 중재시술 치료가 할 수 없는 경우에는 연구에 포함되지 않을 것입니다. 풍선 혈관 확장술로 충분히 치료되어 스텐트를 삽입할 필요가 없는 경우에는 연구에 포함되지 않을 것입니다. 중재시술 치료보다 수술적인 우회술이 환자에게 유익한 경우에는 연구에 포함되지 않을 것입니다.

**새로운 정보 제공 여부**

**We will provide new information**

피험자가 계속적인 임상연구 참여를 결정하거나 연구 진행 과정 동안에 새로이 밝혀지거나 증명되는 모든 의학적 새로운 정보는 연구기간 동안 정보가 입수되는 대로 제공할 것입니다.

**보험 및 보상**

**What if I get hurt during participation in this study?**

중재 시술 및 중재 시술 후 추적검사 시 부담하게 되는 비용은 일반적인 표준치료 및 추적검사에서 부담하게 되는 비용과 같으며 이 연구에 참여함으로써 피험자에게 추가 발생되는 비용은 없습니다. 본 연구는 일반적인 임상에서 시행되는 범위 내에서 이루어지므로 따로 보험 및 보상에 대해서는 규정하지 않습니다.

**피험자의 연구 참여에 대한 보상(이점) (예: 교통비 제공)**

**Will I be paid by participation?**

연구에 참여함으로써 직접적인 금전적인 보상은 없습니다. 하지만 여러분께서 참여하여 주신 연구의 정보를 이용하여 더 나은 치료에 이용될 수 있습니다.

**비밀보장 및 허용 범위**

**Will my personal information be kept confidential?**

모니터요원, 점검을 실시하는 자, 심사위원회 및 식품의약품안전청장은 피험자의 비밀보장을 침해하지 않고 관련규정이 정하는 범위 안에서 임상시험의 실시절차와 자료의 신뢰성을 검증하기 위해 본인의 의무기록을 직접 열람할 수 있으며 본 동의서에 서명함은 이러한 자료의 직접 열람을 허용한다는 것을 의미하며, 임상시험의 결과가 출판될 경우 피험자의 신원은 비밀 상태로 유지될 것이라는 사실을 알려드립니다.

**자유로운 참여 동의와 동의 철회 가능성 및 연구 조기 중단 시 다른 치료방법 및 관련사항**

**The Right of declining to participate or withdraw**

귀하께서 이 연구에 참여하지 않으셔도 되며, 연구에 참여하시지 않더라도 환자는 이로 인해서 의료진이나 병원으로부터 조금의 불이익도 받지 않을 것이며, 저희들은 최선을 다해서 치료를 하겠습니다. 연구에 참여하였다 하더라도 언제든지 취소하실 수 있습니다. 본 연구에 참여하지 않으신 경우에도 통상적인 치료는 적절하게 시행될 것입니다. 본 연구에 대한 동의를 철회하는 경우나 연구자 및 연구 의뢰자에 의해 연구를 조기에 중단할 심각한 문제가 발생하는 경우에는 연구 참여자에게 통고 후 연구 참여를 중지할 수 있습니다.

**임상연구 도중 피험자의 임상연구 참여가 중지되는 경우 및 해당 사유**

연구자는 주기적으로 각각의 환자 군에 대한 안정성 및 이상반응 등을 관찰할 예정이며 예기치 못한 합병증이나 부작용이 지속적으로 문제가 되는 경우 임상시험 도중 피험자의 임상시험 참여를 중지시킬 수 있습니다.

**의뢰 회사**

본 연구는 의뢰자 주도 연구가 아닌 연구자 주도 연구이며 따라서 의뢰 회사는 없습니다.

**피험자 설명문 및 동의서 사본 1부 제공**

환자분께 본 임상 연구에 대한 설명을 잘 듣고 동의하실 경우 환자분에게 피험자 설명문 및 동의서 사본 1부를 보관을 위하여 첨부해 드립니다.

**문의 (Who can I contact, if I have further question?)**

| 본 임상시험과 관련하여 귀하의 권익에 관하여 추가적인 정보를 얻고자 하는 경우 귀하는 고려대학교 의과대학 구로병원 임상시험심사위원회 02) 2626-1106에 연락할 수 있습니다. 만일 귀하가 본 임상시험과 관련하여 이해가 잘 안되는 부분이 있거나 임상시험의 참여 때문에 손상을 입었다면 아래 연락처로 언제든지 연락할 수 있습니다.  시험책임자 : 나 승 운  소속: 고려대학교 의과대학 구로병원 순환기내과  연락처 : 02-2626-1106 |
| --- |

**피험자 동의서**

**Informed Consent Statement**

본인은 ‘동맥 경화성 장골 동맥 동맥 질환에서 자가 팽창형 니티놀 스텐트인 COMPLETE SE^TM^ 스텐트와 풍선 확장형 SCUBA^TM^ 스텐트가 일차적 개존률에 미치는 영향을 평가하기 위한 전향적, 다기관 공동, 무작위 배정 비교 연구’에 관한 임상시험의 목적 및 방법, 기대효과, 가능한 위험성, 타 치료 방법의 내용과 안전대책 및 비밀보장에 대하여 충분한 설명을 듣고 이해하였으며, 모든 궁금한 사항에 대하여 충분한 답변을 들었습니다. 본인은 본 연구에 동의한 경우라도 언제든지 철회할 수 있음과 이에 따른 다른 적절한 치료를 계속 받을 수 있음을 확인하였습니다. 또한 본인은 임상시험 참여자로서의 권리와 의무를 잘 이해하고 있으며 성심껏 협조할 것입니다.

이에 본인은 충분한 시간을 갖고 생각한 이후에 상기 임상시험에 참여하기를 자유로운 의사에 따라 동의하며, 피험자 설명문 및 동의서 사본 1부를 받을 것임을 알고 있습니다.

년 월 일 (date)

| 피험자  Participant | 성 명 | (서명)  (signature) |
| --- | --- | --- |

☞ 피험자의 자필로 서명해야 함

| 시험자  Investigator | 성 명 | (서명)  (signature) |
| --- | --- | --- |
|  | 연락처 | TEL : |
|  |  | H.P : |

☞ 피험자에게 서면동의를 받는 시점에 기입하며 동의서를 설명한 사람 .(시험책임자, 공동연구자, 시험담당자)의 자필로 서명해야 함.
