# Supplementary material for: Study design and rationale of the ‘Balloon-Expandable Cobalt Chromium SCUBA Stent versus Self-Expandable COMPLETE-SE Nitinol Stent for the Atherosclerotic ILIAC Arterial Disease (SENS-ILIAC Trial) Trial’: study protocol for a randomized controlled trial
Source: Trials. 2016 Jun 25;17:302. doi: 10.1186/s13063-016-1435-9 (PMC4920989; doi:10.1186/s13063-016-1435-9)
Supplement: Additional file 3: — The schedule of enrollment, interventions, and assessments. (DOCX 22 kb) [file 13063_2016_1435_MOESM3_ESM.docx]

Table 1. Balloon-Expandable Cobalt Chromium SCUBA Stent versus Self-Expandable COMPLETE-SE Nitinol Stent for the Atherosclerotic ILIAC Arterial Disease (SENS-ILIAC) Trial timeline chart

|  | **STUDY PERIOD** | | | | | |
| --- | --- | --- | --- | --- | --- | --- |
|  | **Enrolment** | **Allocation** | **Post-allocation** | | | **Close-out** |
| **TIMEPOINT** | ***V1*** | **V2** | ***V3*** | ***V4*** | ***V5*** | ***V6*** |
| **ENROLMENT:** |  |  |  |  |  |  |
| Eligibility screen | X |  |  |  |  |  |
| Informed consent | X |  |  |  |  |  |
| *Demographic details and Medical histroy* | X |  |  |  |  |  |
| Allocation |  | X |  |  |  |  |
| **INTERVENTIONS:** |  |  |  |  |  |  |
| Self expandable stent |  |  |  |  |  |  |
| Balloon expandable stent |  |  |  |  |  |  |
| **ASSESSMENTS:** |  |  |  |  |  |  |
| Stent patency |  |  |  | X | X | X |
| Geographic miss and stent fracture |  |  |  | X | X | X |
| Adverse events |  |  | X | X | X | X |
